# Real-time whole-genome sequencing surveillance for outbreak detection and intervention

**DOI:** 10.1017/ash.2023.239

**Published:** 2023-09-29

**Authors:** Alexander Sundermann, Marissa Griffith, Vatsala Rangachar Srinivasa, Kady Waggle, Jane Marsh, Daria Van Tyne, Ashley Ayres, Lora Pless, Graham Snyder, Lee Harrison

## Abstract

**Background:** Detecting healthcare-associated transmission and outbreaks often relies on reactive whole-genome sequencing (WGS), which occurs after the suspected transmission has occurred. Additionally, reactive WGS frequently misidentifies transmission and misses transmission when it has occurred. We initiated weekly real-time WGS to detect bacterial transmission and direct infection prevention interventions. We describe our experience after 1 year of real-time WGS surveillance at the University of Pittsburgh Medical Center–Presbyterian Hospital, a large, tertiary-care facility. **Methods:** Weekly WGS surveillance was performed from November 1, 2021, to October 31, 2022. Cultured isolates of select bacterial pathogens from patients who were hospitalized for ≥3 days or had a recent healthcare exposure in the prior 30 days were collected and sequenced. Isolates that were ≤15 single-nucleotide polymorphisms (SNPs) were considered genetically related clusters except for *Clostridioides difficile* (≤2 SNPs). Genetically related clusters were investigated for epidemiological links and interventions to interrupt transmission were implemented at the discretion of the infection prevention team. We analyzed subsequent infections that occurred within an outbreak after an intervention was in place. **Results:** In total, 1,909 isolates were sequenced. Of 1,633 unique patient isolates clustered by sequence type, 74 clusters were identified comprising 210 (12.9%) patient isolates (Table 1). The median time from culture date to sequencing was 14 days (IQR, 5.25). The median cluster size was 2 (IQR, 1) (Table 2). Overall, 118 patient isolates (56.2%) had an epidemiological link to a prior isolate, indicating potential transmission. Of 74 clusters, 66 (89.2%) received infection prevention interventions after notification based upon epidemiological data. The infection prevention team performed 69 total interventions, which included unit education (n = 28), hand hygiene observations (n = 16), enhanced cleaning (n = 16), environmental cultures or removal of endoscope (n = 7), and enhanced microbiology surveillance (n = 2). The 59 subsequent infections after infection prevention notification included 17 (28.8%) with no clear epidemiological link, and 41 (69.5%) with an epidemiological link either to a new transmission route (n = 37) or the same route prior to infection prevention intervention (n = 4). Only 1 (1.7%) subsequent infection within a cluster occurred after an infection prevention intervention from the same potential route, which was a suspected unit-based transmission of vancomycin-resistant *Enterococcus faecium*. **Conclusions:** Real-time WGS was effective at detecting genetically related clusters, finding potential sources, and halting further transmission after interventions by the infection prevention team. Quick turnaround times from patient culture to sequencing and analysis were vital for successful WGS surveillance. Real-time WGS surveillance has the potential to substantially shift the infection prevention paradigm for outbreak detection.

**Figure:**
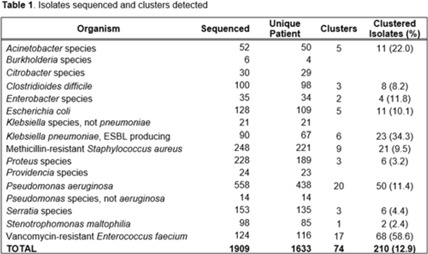

**Figure:**
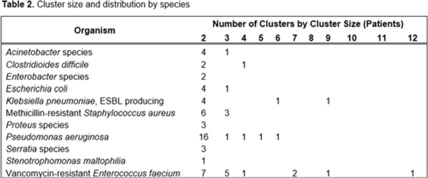

**Disclosure:** None

